# Lipid-associated macrophages and metabolic inflammatory diseases

**DOI:** 10.1016/j.cellin.2026.100312

**Published:** 2026-02-27

**Authors:** Yuxiao Zheng, Dezhen Tu, Chen Dong, Haiqing Zhao, Hongyan Wang

**Affiliations:** a Key Laboratory of Systems Health Science of Zhejiang Province, School of Life Science, Hangzhou Institute for Advanced Study, University of Chinese Academy of Sciences, Hangzhou, 310024, Zhejiang, China; b Innovative Institute of Tumor Immunity and Medicine, Anhui Province Key Laboratory of Tumor Immune Microenvironment and Immunotherapy, The First Affiliated Hospital of Anhui Medical University, Hefei, 230022, Anhui, China; c State Key Laboratory of RNA Innovation, Science and Engineering, Shanghai Institute of Biochemistry and Cell Biology, Center for Excellence in Molecular Cell Science, Chinese Academy of Sciences, University of Chinese Academy of Sciences, Shanghai, 200031, China

**Keywords:** Lipid-associated macrophage, Lipid disorders, Immunometabolism, Inflammation-related disease

## Abstract

Lipid metabolism disorders have been identified in various chronic diseases that are also closely linked with immune cells and inflammatory microenvironment. As pivotal innate immune cells, macrophages exhibit remarkable heterogeneity. Lipid-associated macrophages (LAMs) have emerged as a distinct subtype, characterized by high expression of lipid metabolism-related genes and abundant intracellular lipid droplets (LDs). Accumulating evidence suggests LAMs at the crossroads of lipid dysregulation and inflammation during the development of various diseases. This review focuses on LAMs in four pathological contexts: the adipose tissue in obesity, the liver in lipid metabolism disorders, atherosclerotic cardiovascular diseases, and the brain in neurodegenerative diseases. The features of LAMs, including specific markers, cellular origins, tissue-specific localization, and their regulatory roles in lipid metabolism, inflammation as well as tissue injury or repair within these pathological microenvironments, are discussed. Additionally, multiple LAM-related molecular targets and relevant clinical trials across different disease conditions are summarized, providing insights for future research and clinical translation in chronic inflammatory diseases.

## 1 Introduction

Lipid metabolism, including lipid synthesis, transportation, storage and clearance, is crucial for maintaining energy balance and physiological function of various types of cells. Dysregulation of lipid metabolism, such as excessive lipid synthesis, impaired breakdown, or abnormal transport, is involved in the development of various chronic diseases (Zadoorian et al., 2023). Also, lipid metabolism disorders link with inflammatory environment in the lesion sites of obesity, metabolic dysfunction-associated steatotic liver disease (MASLD)/metabolic dysfunction-associated steatohepatitis (MASH), neurodegenerative diseases, atherosclerosis (Chen et al., 2019; Gupta et al., 2020; Mato et al., 2019; Yang et al., 2023).

Macrophages are widely distributed in tissues and organs, playing a critical role in immune defense, tissue repair, or the development of tumors, autoimmune diseases or metabolic diseases. Macrophages were initially divided into classically activated (pro-inflammatory M1) and alternatively activated (anti-inflammatory M2) subsets (Orecchioni et al., 2019; Yunna et al., 2020). With single-cell RNA sequencing and advanced technologies of flow cytometry, the heterogeneity of macrophages has been revealed, including various tissue-specific and functionally specialized macrophage subpopulations. Lipid-associated macrophages (LAMs) are recently identified macrophage subtypes in adipose tissue, characterized by high expression of genes related to lipid metabolism and abundant LDs within the cells (Jaitin et al., 2019). This article focuses on LAMs characteristics, regulatory mechanisms and pathological effects in fat, liver, cardiovascular system and brain related diseases, which might help us to reveal new targets for the treatment of obesity, cardiovascular diseases, neurodegenerative diseases (Jaitin et al., 2019; Marschallinger et al., 2020; Remmerie et al., 2020).

## 2 Overview of LAMs

LAMs represent a specialized subset of macrophages characterized by abundant intracellular LDs, robust capacities for lipid phagocytosis, storage and metabolism, as well as pivotal roles in immune regulation (Xu et al., 2024). LAMs arise either from the lipid-stimulated reprogramming of tissue-resident macrophages or from the recruitment and differentiation of circulating monocytes in response to local tissue lipid overload. In adipose tissue, LAMs typically localize around hypertrophic or dying adipocytes, forming characteristic crown-like structures (CLS) that facilitate direct contact with adipocytes and efficient phagocytosis of excess or released lipids. The identification of LAMs relies on distinct surface markers, which can be categorized into three classes: (i) lipid-sensing receptors responsible for detecting lipid signals (e.g., TREM2 and CD36); (ii) lipid metabolic molecules involved in lipid degradation, transport and storage (e.g., APOE and PLIN2); (iii) inflammatory and reparative molecules associated with immune regulation (e.g., CD163 and SPP1) (Xu et al., 2024). The combinatorial expression of these markers distinguishes LAMs from conventional M1/M2 macrophages classifications. Functionally, LAMs act as key regulators of tissue lipid stress, and their biological roles are highly context-dependent, varying with tissue microenvironment and disease stage. Consequently, LAMs function as a double-edged sword in metabolic homeostasis, inflammatory regulation and disease progression (Xu et al., 2024).

Of note, adipose tissue macrophages (ATMs) and LAMs represent two closely related yet distinct macrophage populations. Conceptually, ATMs refer to all macrophages residing in white or brown adipose tissue, irrespective of their phenotype, metabolic status, or function (Nance et al., 2022). In contrast, LAMs constitute a functionally defined subset that present across multiple tissues, occurring not only in adipose tissue but also widely in atherosclerotic plaques, fatty liver, brain, and other organs. In terms of molecular identity, ATMs express a broad panel of myeloid/macrophage markers, such as CD45, F4/80, and CD11b, without a unified signature indicative lipid metabolic specialization (Matz et al., 2023). With respect to inducing factors, ATM accumulation and activation are highly dependent on the adipose tissue microenvironment, including adipocyte hypertrophy and necrosis, spillage of free fatty acids, local inflammatory cytokines, insulin resistance, hypoxia, lipotoxicity, and the need for tissue remodeling and debris clearance (Li et al., 2023a; Nance et al., 2022). Metabolically, ATMs display substantial heterogeneity, with markedly distinct metabolic profiles that closely correlate with homeostatic or polarized states (Li et al., 2023b). Therefore, ATMs fulfill a broad range of immune and homeostatic demands in adipose tissue (Chakarov et al., 2022). In comparison, the inducing stimulus for LAMs is lipid overload, which is independent of tissue type. LAMs exhibit a relatively conserved metabolic program characterized by enhanced lipid uptake and catabolism, with consistent features observed across tissues. Functionally, LAMs are narrowed to mitigate lipid stress. In summary, ATMs are defined primarily by spatial localization within adipose tissue with diverse functions, whereas LAMs are defined by phenotypic identity in response to lipid overload.

## 3 LAMs in the adipose tissue of obese individuals

Adipose tissue is the main site for energy storage, which is also an important endocrine and immune organ (Silva and Baptista, 2019; Unser et al., 2015). When the energy intake of the body is consistently higher than the energy consumption, the excess energy will be converted into lipids and accumulate in adipocytes. The distribution of adipocytes within adipose tissue exhibits distinct regional variations (Yang Loureiro et al., 2022). Mature adipocytes aggregate in lobules. Within these adipose lobules, mature adipocytes coexist with adipose-derived stem cells, macrophages, and other cell types. The primary white adipocytes in humans are categorized into two main types: subcutaneous and visceral. Subcutaneous adipose tissue is predominantly located between the dermis and the muscle layer. Visceral adipose tissue is concentrated in areas such as the greater omentum, mesentery, and perirenal regions, and its excessive accumulation is closely associated with metabolic risks. Brown adipocytes are primarily distributed in the cervicothoracic region and can be activated by cold exposure to generate heat. Beige adipocytes are interspersed within subcutaneous white adipose lobules; they can be induced by external stimuli and possess both energy storage and thermogenic functions. Dysfunction of adipose tissue is closely related to various metabolic diseases, such as obesity, type 2 diabetes and cardiovascular diseases. In the obese state, the expansion of adipose tissue is not only a change in the number and volume of adipocytes, but also accompanied by the imbalance of lipid droplet (LD) homeostasis and local inflammatory responses (Blüher, 2020).

Previous research has focused primarily on inflammatory macrophages in obese states, such as M1 macrophages (Zhou et al., 2025b). However, with advancements in technologies such as single-cell RNA sequencing, a distinct subtype with common macrophage markers *Lyz2*, *Adgre1* (F4/80), and *Cd68*, is characterized by high expression of lipid metabolism-related genes, including *Trem2*, *Cd9*, *Lpl*, and *Cd36*, as well as abundant LDs within their cytoplasm (Cottam et al., 2022; Jaitin et al., 2019; Reyes-Farias et al., 2025). These ATMs, designated as LAMs, have been identified in both mouse and human adipose tissue (Cottam et al., 2022; Jaitin et al., 2019; Reyes-Farias et al., 2025). Notably, the expression profiles of LAMs do not strictly overlap with traditional M1/M2 macrophage markers and are distinctly different from tissue-resident macrophages (TRMs) and circulating macrophages (Jaitin et al., 2019; Nawaz et al., 2023). In both high-fat diet (HFD)-induced obese mouse model and genetic *db/db* mouse model, these macrophages account for more than 75% of myeloid cells in visceral white adipose tissue (Jaitin et al., 2019; Stansbury et al., 2023).

In both obese mice and humans, LAMs are primarily localized in CLS, which form around individual dead adipocytes (Jaitin et al., 2019; Lempicki et al., 2024). In contrast, the non-LD-enriched ATMs are evenly distributed among adipocytes (Hill et al., 2018). During obesity, adipocytes undergo significant cell death due to excessive hypertrophy (Cinti et al., 2005). Dead adipocytes release chemotactic signals, such as free LDs and cellular debris, which recruit monocytes from circulation into adipose tissue, where they differentiate into macrophages (Jaitin et al., 2019; Odegaard and Chawla, 2013). These macrophages recognize dead adipocytes through molecules like MAC-2 and form CLS around them (Molinaro et al., 2017). Simultaneously, they become activated and express inflammatory molecules, such as TNF-α (Cinti et al., 2005). Regions of high LAM concentrations are also enriched with genes related to autophagy and cell death, including *Ctsl*, *Ctss*, *Lamp1*, and *Ctsd*, participating in the clearance of excess lipids and dead adipocytes (Cinti et al., 2005; Stansbury et al., 2023; Wundersitz et al., 2022). Most of these macrophages that emerge in the obese state originate from circulating monocytes (Jaitin et al., 2019; Stansbury et al., 2023). In comparison, adipose TRMs, some of embryonic origin, are already present in the adipose tissue of mice on a normal diet (Jaitin et al., 2019). During their differentiation process, adipose TRMs gradually lose the expression of genes such as *Ly6c2* and *Ccr2* while acquiring expression of genes related to lipid metabolism and phagocytosis, such as *Trem2*, *Lipa*, and *Lpl* (Carrera et al., 2023; Cottam et al., 2022; Stansbury et al., 2023).

**Fig. 1:**
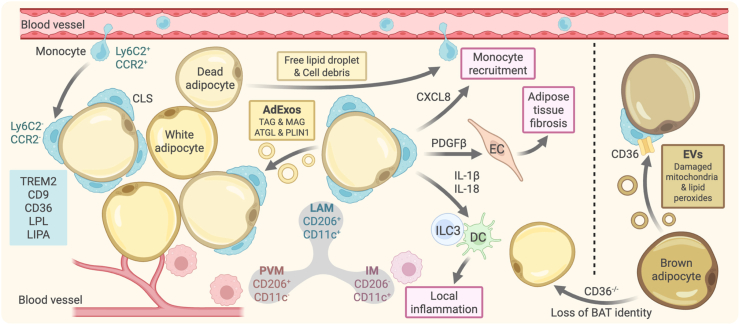
**Origin and functional regulation of LAMs in adipose tissue** Peripheral Ly6C2^+^CCR2^+^ monocytes enter adipose tissue, lose expression of Ly6C2 and CCR2, and differentiate into LAMs. LAMs are characterized by markers such as TREM2, CD9, CD36, LPL, and LIPA, to form CLS around dead adipocytes. Dead adipocytes release AdExos that are taken up by LAMs. LAMs secrete CXCL8 to recruit peripheral monocytes; PDGFβ released by LAMs induces endothelial cells to promote adipose tissue fibrosis; IL-1β and IL-18 secreted by LAMs stimulate ILC3s and DCs to amplify local inflammation; Brown adipocytes release exosomes containing damaged mitochondria and lipid peroxides, which are taken up by LAMs via CD36; and macrophage-specific deletion of CD36 promotes the loss of brown adipocyte identities. Created in BioRender.com. AdExos: adipocyte-derived exosomes. ATGL: adipose triglyceride lipase. BAT: brown adipose tissue. CCR2: C-C chemokine receptor type 2. CD9/36/11c/206: cluster of differentiation 9/36/11c/206. DC: dendritic cell. EC: endothelial cell. EVs: extracellular vesicles. CXCL8: C-X-C motif chemokine ligand 8. IL-1β/18: interleukin-1 beta/18. ILC3: Group 3 innate lymphoid cell. LAM: lipid-associated macrophage. LIPA: lipase A. LPL: lipoprotein lipase. Ly6C2: lymphocyte antigen 6C2. MAG: monoacylglycerol. IM: inflammatory macrophage. PDGFβ: platelet-derived growth factor beta. PLIN1: perilipin 1. PVM: perivascular macrophage. TAG: triacylglycerol. TREM2: triggering receptor expressed on myeloid cells.

TREM2 serves as a core marker for LAMs, which has been identified as a profound immune regulatory receptor in myeloid cells (Jaitin et al., 2019; Ulland and Colonna, 2018). TREM2 plays a pivotal role in microglia to regulate phagocytosis, survival, and proliferation, suppressing excessive inflammatory responses, and maintaining central nervous system homeostasis. Recently, TREM2 is also implicated in lipid sensing and metabolism to regulate functions of LAMs. *Trem2*^−/−^ obese mice exhibit a loss of LAM gene signatures, which show impaired lipid uptake and storage functions, exacerbated adipocyte hypertrophy, accelerated weight gain, and metabolic abnormalities such as glucose intolerance. It indicates that *Trem2*^+^ LAMs might play a protective role in lipid regulation during obesity. In human white adipose tissue (WAT), LAMs are identified as a subset of CD206^+^CD11c^+^ macrophages, distinguishable from CD206^+^CD11c^-^ perivascular macrophages (PVM) and CD206^−^CD11c^+^ inflammatory macrophages (IM) based on specific markers (Hildreth et al., 2021; Nance et al., 2022) (Fig. 1). Their molecular markers TREM2, CD9, and LPL are highly conserved between humans and mice and enrich in subcutaneous WAT, serving as potential markers of obesity-related inflammation in adipose tissue. The proportion of LAMs in WAT from obese individuals is significantly higher than in lean controls, which correlates positively with body mass index (BMI). LAMs are also important sources of local pro-inflammatory factors such as interleukin-1β (IL-1β), TNF-α, IL-18, CXCL8, and PDGFβ that interact with structural and immune cells. These interactions regulate adipose tissue fibrosis and promote inflammatory responses, with the recruitment of monocytes, establishing a positive feedback loop of “monocyte recruitment-LAM differentiation-exacerbated inflammation”.

In the visceral fat of obese patients and mice, CD9^+^ ATMs, belonging to the CD11b^+^Ly6C2^−^CD64^+^F4/80^+^ macrophage subset, are characterized by high LD content within their lysosomal structures (Hill et al., 2018). In contrast, CD9^−^ ATMs and Ly6C2^+^ ATMs contain minimal LDs. The expression of CD9 in CD9^+^ ATMs increases with the severity of obesity induced by an HFD. In human patients, CD9 expression also positively correlates with BMI. This macrophage subset exhibits unique transcriptional characteristics associated with pro-inflammatory and lysosomal lipid metabolism: expressing high levels of pro-inflammatory genes such as *Ccl2*, *Il1a*, *Il18*, and *Tnf*, and lysosomal lipid metabolism genes such as *Acp5*, *Ctss*, *Lamp2*, and *Lipa*, while showing low expression of genes related to angiogenesis and tissue homeostasis (Caslin et al., 2020). CD9^+^ ATMs *in vivo* can directly induce a phenotype of obesity-related inflammation in normal adipose tissue (Arivazhagan et al., 2020). Their lysosomal LD metabolism may help clear lipids released from dead adipocytes, a process that is accompanied by inflammatory responses (Jaitin et al., 2019).

The number of LAMs in the epididymal fat of obese mice increases and exhibits a pro-inflammatory phenotype, positively correlating with LD accumulation and the expression of T cell exhaustion markers PD-1 and TIGIT (Cottam et al., 2022). This suggests LAMs may contribute to T cell dysfunction via antigen presentation or cytokine secretion. Following weight loss, the number of LAMs does not return to levels observed in lean mice; the pro-inflammatory phenotype and certain obesity-related gene expression patterns persist, while lipid-related functions are delayed in recovery (Weinstock et al., 2019). After weight regain, the number of LAMs increases significantly in the obese group, along with exacerbated inflammation (Schmitz et al., 2016). Additionally, intermittent fasting (IF) exacerbates the inflammatory phenotype of LAMs in visceral fat of obese mice, with LAM accumulation depending on p53 in adipocyte (Reinisch et al., 2024). Specific knockout of p53 in adipocytes can prevent IF-induced LAM accumulation and improve lipolysis, systemic metabolic flexibility, and insulin sensitivity. The expression of TREM2 and p53 in the visceral fat of the weight regain group is significantly higher compared to that in the weight loss group, and TREM2 expression correlates positively with the rate of BMI regain. This suggests that targeting p53 in LAMs may improve weight loss outcomes (Reinisch et al., 2024).

The storage of neutral lipids in ATMs significantly differs from other macrophages. Typically, neutral lipids in general macrophages are stored within conventional LDs, and these LDs can be transported to lysosomes for degradation via autophagy (Flaherty et al., 2019; Kimmel and Sztalryd, 2016; Ouimet et al., 2011; Stoffel et al., 2021; Straub et al., 2013). In contrast, ATMs exhibit almost no autophagolysosomal structures; instead, they store lipids in vesicles rather than in traditional LDs (Flaherty et al., 2019). These lipid vesicles are formed by ATMs internalizing adipocyte-derived exosomes (AdExos) released by adipocytes. AdExos contain neutral lipids such as triglycerides and adipocyte-specific proteins, mediating a local “adipocyte-ATM” lipid recycling circuit (Flaherty et al., 2019; Nielsen et al., 2014; Storlien et al., 2004). This circuit is crucial for maintaining adipose tissue homeostasis. Mice lacking the ability of ATMs to hydrolyze triglycerides in endolysosomes exhibit the atrophy of fat depots (Flaherty et al., 2019).

## 4 LAMs across obesity models and adipose tissue

LAMs exhibit model- and tissue-specific differences. In mouse models, LAMs in *db/db* mice predominantly engage in mitochondrial oxidative phosphorylation, whereas LAMs in HFD-induced obese mice are characterized by upregulated fatty acid metabolism and lysosomal activity (Ninni et al., 2025). In the brown adipose tissue (BAT) of *db/db* mice, LAMs progressively increase with obesity progression and express the lipid scavenger receptor CD36 (Sciarretta et al., 2024). CD36 mediates the uptake of lipids and oxidized low-density lipoprotein (oxLDL), participating in foam cell formation, inflammatory responses, phagocytic clearance, and metabolism regulation, which is crucial for maintaining cellular lipid homeostasis and innate immune response (Wang and Li, 2019). Metabolically stressed brown adipocytes release extracellular vesicles containing damaged mitochondria and lipid peroxides, LAMs capture these vesicles via CD36, acquiring a foam cell-like phenotype (Ninni et al., 2025; Sciarretta et al., 2024) (Fig. 1). Additionally, mice with macrophage-specific CD36 knockout exhibit upregulated mitochondrial oxidative function genes but reduced mitochondrial quality in BAT. Furthermore, LAMs can induce WAT-associated genes such as *Aldh1a1* in BAT through TGF-β1, inhibiting mitochondrial-related genes and promoting a transition to a WAT-like phenotype (Sciarretta et al., 2024). A *Pparg*^high^ LAM subpopulation is characterized by active PPAR signaling pathways and exists in BAT (Itoh et al., 2008; Ninni et al., 2025; Odegaard et al., 2008). In HFD-induced obese mice, the loss of myeloid PPAR-γ leads to the loss of thermogenic characteristics in BAT and reduced expression of UCP1, manifesting a WAT-like phenotype. Conversely, LAMs in the BAT of *db/db* mice directly restricts thermogenesis in mature brown adipocytes (Ninni et al., 2025).

In summary, LAMs act as a core subset to regulate adipose tissue microenvironmental homeostasis under obesity conditions. LAMs in subcutaneous versus visceral fat, or in brown versus white adipose tissue, show different molecular expression and functional characteristics. For example, LAMs in HFD-induced and genetic obesity models display distinct metabolic and functional profiles; their interaction with adipose tissue persists throughout the entire developmental process of obesity-related metabolic disorders. It is critical to further elucidate adipose tissue and LAM interactions, including their distinct roles in metabolic processes such as lipid uptake, storage, and clearance, as well as in initiating and exacerbating local adipose tissue inflammation via the secretion of pro-inflammatory factors. In addition, an in-depth analysis is required to characterize the heterogeneity of LAMs in different adipose depots, including gene markers, differentiation or activation processes, metabolic signaling pathways, and intercellular interactions. Such investigations might help to precisely target LAMs and develop novel therapeutic strategies for obesity and related metabolic diseases. For example, activating the core marker TREM2 can enhance the lipid-regulatory function of LAMs (Jaitin et al., 2019); inhibiting p53 can reduce abnormal LAM accumulation and improve weight loss outcomes (Schmitz et al., 2016); targeting CD36 can block the formation of foam cell-like phenotypes in LAMs and maintain the thermogenic properties of brown adipose tissue, while downregulating pro-inflammatory gene expression can break the inflammatory positive feedback loop (Sciarretta et al., 2024).

## 5 LAMs in lipid metabolism-related liver disorders

The liver serves as the central organ for lipid metabolism, and its metabolic homeostasis is crucial for maintaining overall lipid balance in the body. Factors such as unhealthy lifestyles and metabolic abnormalities contribute to a rising prevalence of MASLD and its progressive stages, including MASH as well as liver fibrosis. MASLD is characterized by excessive fat accumulation in hepatocytes, following liver injury such as alcohol abuse, viral hepatitis, and drug-induced liver damage. MASH, in addition to steatosis, is accompanied by significant hepatocyte inflammation, necrosis, and ballooning degeneration. Liver fibrosis is a reparative overgrowth response following chronic liver injury. MASLD is a significant predisposing factor for liver fibrosis, and MASH carries a much higher risk of progressing to fibrosis than MASLD due to persistent inflammatory damage (Kumar et al., 2021; Powell et al., 2021; Sheka et al., 2020). Macrophage subpopulations in the liver microenvironment are heterogeneous, comprising liver-resident Kupffer cells (KCs) located in the hepatic sinusoids and monocyte-derived macrophages derived from bone marrow hematopoietic stem cells (De Simone et al., 2021; Huang et al., 2024).

LAMs in the liver are derived from monocytes expressing high levels of *Ly6c2* and *Ccr2*, rather than through the proliferation of resident KCs (ResKCs) (Daemen et al., 2021; Fredrickson et al., 2025; Guo et al., 2024; Remmerie et al., 2020; Wang et al., 2024; Xu et al., 2025b). This indicates the presence of three distinct macrophage populations in the liver: ResKCs, monocyte-derived KCs (moKC), and monocyte-derived LAMs (Remmerie et al., 2020). LAMs are predominantly localized in regions containing fibrotic stellate cells and myofibroblasts, and are more concentrated in areas where ResKCs are progressively lost during the progression of MASLD, such as the hepatic periportal zone (Ganguly et al., 2024; Li et al., 2025b; Xu et al., 2025a; Zhang et al., 2018). Their distribution shows substantial spatial overlap with steatotic lesions, suggesting a close association between LAMs and the fibrotic liver microenvironment. LAMs also predominantly accumulate within hepatic CLSs (hCLSs) surrounding steatotic hepatocytes (Daemen et al., 2021; Ioannou et al., 2013; Ioannou et al., 2015; Xu et al., 2025a). The formation of these structures depends on a transitional *Cx3cr1/Ccr2*-expressing LAM subset (C-LAM). Knockout of *Ccr2* leads to a reduction in C-LAMs, impaired hCLS formation, increased hepatic collagen deposition, and aggravated fibrosis (Daemen et al., 2021). The distribution and abundance of LAMs dynamically change with the course of MASH: their numbers are lower in the progression phase, and no new macrophage subsets are generated during the resolution phase; however, macrophage composition is reconstituted, with LAMs becoming the dominant subset. Mature LAMs do not express *Cx3cr1/Ccr2* but highly express *Cd63* and *Gpnmb*. It is hypothesized that LAMs may reduce dysregulated inflammation and fibrosis by clearing necrotic steatotic hepatocytes, phagocytosing toxic lipids, or suppressing excessive stellate cell activation (Daemen et al., 2021; Deng et al., 2026).

Upon being fed with a western diet (WD), CLEC4F^-^ macrophage subset in the livers of mice display a similar gene expression profile as that of LAMs in obese adipose tissue, including enrichment in LAM markers such as *Trem2*, *Cd9*, *Spp1*, *Fabp5*, and *Gpnmb*, while expressing low levels of KC-specific markers like *Timd4*, *Clec4f*, *Marco*, and *Cd163* (Daemen et al., 2021; Fredrickson et al., 2025; Guo et al., 2024; Jaitin et al., 2019; Wang et al., 2024; Xu et al., 2025a; Xu et al., 2025b). Nevertheless, differences exist between LAMs and CLEC4F^+^ ResKCs/moKCs: certain phospholipids, triglycerides as well as apoptosis-related genes are detected at higher levels in CLEC4F^+^ cells, compared to LAMs. Consistently, LAMs exhibit a longer survival time in the liver, approximately 1-2 weeks (Guo et al., 2024; Remmerie et al., 2020).

In mouse models of MASLD induced by an HFD or the amylin liver non-alcoholic steatohepatitis (AMLN) diet, *Trem2* expression levels are elevated in LAMs compared to those in normal diet control groups (Remmerie et al., 2020; Wang et al., 2024). TREM2 can regulate LAM function in the liver. LAMs inhibit NLRP3 inflammasome activation via TREM2 signaling, thereby reducing the release of pro-inflammatory cytokines IL-1β and TNF-α (Ganguly et al., 2024; Zhou et al., 2025a). Loss of TREM2 not only decreases LAM abundance and impairs hCLS formation, but also directly disrupts their functions including phagocytosis, metabolic adaptation, and inflammatory regulation (Ganguly et al., 2024). Vertical sleeve gastrectomy (VSG) enhances the efferocytosis and lipid-handling capacity of LAMs in MASH mice (Cherla et al., 2020; Fredrickson et al., 2025; Özer et al., 2018). This effect is also linked to the surface receptor TREM2 on LAMs: in *Trem2*-knockout mice undergoing VSG, the reparative properties of LAMs are abrogated, and MASH-related pathological manifestations fail to improve. Under MASH conditions, TREM2-deficient LAMs exhibit an imbalance between pro-inflammatory and reparative responses. Following VSG intervention, LAMs shift toward an anti-inflammatory phenotype, which aligns with the observed reduction in LAM-associated inflammatory gene expression in human MASH patients after surgery (Ahn et al., 2021; Biobaku et al., 2020; Fredrickson et al., 2025). In human MASH livers, macrophages are enriched with *TREM2* and *MS4A7* expression, and *MS4A7* expression was positively correlated with *TREM2* and *COL1A1* expression (Zhou et al., 2024). In MASH mice, the expression of *Ms4a7* also showed a strong positive correlation with *Trem2* expression and plasma alanine aminotransferase (ALT) concentration (Zhou et al., 2024); an both expressions decreased after switching to a standard chow diet. These findings suggest that TREM2 and MS4A7 may serve as potential biomarkers for MASH progression.

Others reported LDs as a key inducer of LAM differentiation. *Ex vivo* experiments also confirmed that LDs could induce bone marrow-derived macrophages (BMDMs) to differentiate into an LAM-like phenotype. LDs derived from hepatocytes in HFD-induced obese mice can be phagocytosed by KCs and monocyte-derived macrophages (MDMs) in the liver (Zhou et al., 2024). Continuous injection of LDs induced the infiltration of F4/80^mid^CD11b^high^ macrophages, upregulating the expression of LAM markers (*Trem2*, *Gpnmb*, and *Ms4a7*), inflammatory cytokines (*Ccl2* and *Tnf*), and NLRP3 inflammasome-related genes (*Nlrp3*, *Pycard*, and *Il1b*). These findings suggest that the link “hepatocyte lipolysis-LD release-macrophage LAM polarization” is critical in the progression of MASH from steatosis to inflammation/fibrosis (Zhou et al., 2024).

The Notch-RBPJ signaling pathway acts as a key switch for monocyte differentiation into LAMs, maintaining LAM numbers while limiting their lipid uptake (Bonnardel et al., 2019; Guilliams et al., 2022; Guo et al., 2024). Targeting this pathway can reduce the generation of pro-inflammatory macrophages, preserve lipid clearance capacity, and alleviate disease progression. And LAMs in MASH patients could secrete hepatocyte growth factor (HGF) to promote hepatocyte proliferation and inhibit apoptosis, exerting a protective role in liver injury repair. Thyroid hormone-inducible hepatic protein (SPOT14), involved in fatty acid synthesis, is significantly elevated in the livers of both MASH patients and mouse models (Xu et al., 2025a). It promotes hepatocyte lipid synthesis and macrophage migration inhibitory factor (MIF) secretion. MIF binds to the CD74 receptor on the LAM surface, recruiting and activating LAMs, ultimately exacerbating hepatic inflammation and fibrosis (Bernhagen et al., 2007; Marin et al., 2017). To simulate the metabolic background of MASH patients, researchers transplanted bone marrow cells from Niemann-Pick type C1 (NPC1) mutant mice into *Ldlr*^−/−^ mice and induced a lipid-accumulating environment rich in LDL and oxLDL using an HFD (Houben et al., 2017). This resulted in higher hepatic cholesterol levels and significantly upregulated expression of lipid transport-related genes. These phenomena are attributed to impaired lysosomal lipid transport in macrophages due to the *Npc1* mutation, leading to lipid accumulation. OxLDL is a key driver of macrophage lipid accumulation, as it stimulates *Npc1*-mutant BMDMs to upregulate the expression of *Tnf* and *Ccr2*, an effect that can be abolished by anti-oxLDL antibodies (Houben et al., 2017).

In summary, LAMs, as a specialized subset derived from bone marrow monocytes, differ from ResKCs and moKCs in terms of origin, gene expression, lipid composition, or function. The regulatory circuit of hepatocyte lipolysis, LD release, and LAM polarization is critical to drive the progression of MASLD from steatosis to inflammation and fibrosis in MASH. LAMs can alleviate dysregulated hepatic inflammation and fibrosis by phagocytosing necrotic steatotic hepatocytes, processing toxic lipids, and suppressing excessive activation of hepatic stellate cells. Furthermore, LAMs regulate inflammatory responses via TREM2 signaling or secreting HGF to promote liver repair. Understanding the origin, differentiation, and function of LAMs at different stages of MASLD/MASH progression might provide targets to improve hepatic lipid metabolic homeostasis, mitigate inflammatory injury, and inhibit the progression of liver fibrosis, thereby developing new strategies for the treatment of MASLD/MASH.

## 6 LAMs in atherosclerosis

Atherosclerosis serves as the core pathological basis for most cardiovascular diseases, which are closely associated with lipid metabolic disorders and immune cell dysfunction. During lipid metabolic disorders, excess LDL cholesterol (LDL-C) is oxidized to form oxLDL, which infiltrates the arterial intima and damages endothelial cells, thereby recruiting monocytes from the blood to migrate into the intima and differentiate into macrophages (Mineo, 2020; Xing and Lin, 2025). Macrophages recognize and phagocytose abnormal lipids such as oxLDL via scavenger receptors SR-A, CD36, and OLR1. Following excessive lipid accumulation, these macrophages transform into lipid-laden macrophages, a hallmark of early plaque formation in atherosclerosis. Among these, foam cells are the most extensively studied subtype (Chistiakov et al., 2017; Wang et al., 2019a). As the primary component of the plaque lipid core, foam cells not only store lipids but also secrete inflammatory factors TNF-α, IL-6, and IL-1β, chemoattractants, and matrix metalloproteinases. It may exacerbate local inflammation, disrupt vascular matrix integrity, and promote plaque proliferation, instability, and even rupture (Chistiakov et al., 2017; Wang et al., 2019a).

**Fig. 2:**
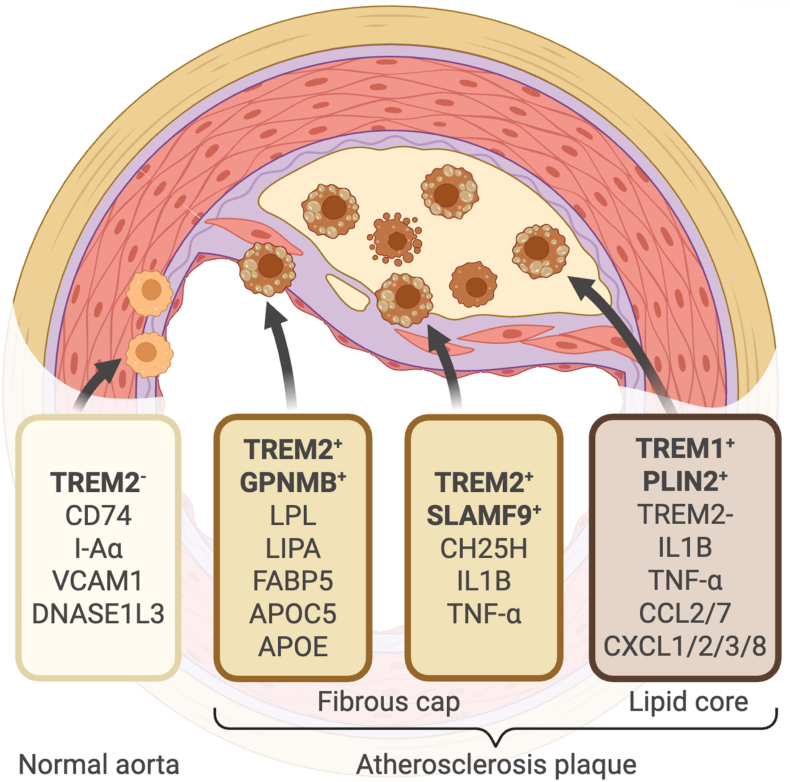
**Characteristics of LAMs in normal aortas vs. atherosclerosis** In normal aortas, LAMs are *Trem2*^-^. LAMs within the fibrous cap of plaques consist of two subtypes, *Gpnmb*^+^ and *Slamf9*^+^, both of which express *Trem2*. LAMs in the rupture-prone plaque core highly express *Trem1* and *Plin2*. Created in BioRender.com.

High-resolution re-clustering of aortic mononuclear phagocytes from three atherosclerotic mouse models (*Ldlr*^−/−,^ *Apoe*^−/−^, and PCSK9-adeno-associated virus induction) identified two LAM subpopulations that highly express the core foam cell marker *Trem2* but exhibit distinct transcriptional profiles (Johnston et al., 2018; Lee and Choi, 2020; May et al., 2022; Zernecke et al., 2023) (Fig. 2). The *Trem2*^high^-*Gpnmb* subtype highly expresses *Gpnmb* and is enriched for lipid metabolism-related genes *Apoc1*, *Apoe Lpl*, *Lipa*, and *Fabp5* (Patterson et al., 2023). GPNMB is a transmembrane glycoprotein involved in the progression of cancer, metabolic disorders, and inflammatory diseases by regulating macrophage function, cholesterol metabolism, and tissue homeostasis (Gong et al., 2019). The apolipoprotein (APO) family is critical for lipid transport and metabolism to maintain lipid homeostasis in the central nervous system and peripheral tissues (Mehta and Shapiro, 2022). This subtype significantly increases after atherosclerosis induction, constituting 38% of intimal BODIPY^high^ foam cells, and represents the core LAMs within lesions (Maisa et al., 2015; Wang et al., 2019b). It shows significant association with plaque calcification and cellular aggregate formation. The *Trem2*^high^-*Slamf9* subtype highly expresses *Slamf9*, and lower levels of inflammation-related genes *Tnf*, *Il1b*, and *Ch25h* than classical pro-inflammatory macrophages (Zernecke et al., 2023). It constitutes 14% of BODIPY^high^ foam cells and represents a transitional subtype characterized by lipid enrichment and mild inflammation. This subtype is enriched for the negative regulation of macrophage colony-stimulating factor (M-CSF) signaling pathway, potentially involved in regulating macrophage proliferation (Woo et al., 2018; Zheng et al., 2018). The inflammatory LAM subtype has been identified by single-cell RNA sequencing of carotid plaque tissues from patients with atherosclerosis who had undergone carotid endarterectomy (Dib et al., 2023; Fernandez et al., 2019; Kim et al., 2018). Conventional *TREM2*^high^ LAMs are predominantly located near the stable fibrous cap of plaques, which highly express *TREM2*, *FABP4/5*, and *CD36*, with enriched cholesterol efflux and lysosomal degradation pathways, displaying a homeostatic lipid-handling phenotype and low levels of inflammatory gene expression (Dib et al., 2023; Fernandez et al., 2019; Zernecke et al., 2023). In contrast, PLIN2^high^/TREM1^high^ LAMs are mainly situated in the rupture-prone lipid core of plaques, which lack genes involved in cholesterol efflux and lysosomal degradation but highly express inflammatory genes (*IL1B*, *TNF*, and *CEBPB*) and chemoattractants (*CCL2/7* and *CXCL1/2/3/8*), therefore presenting a dual “inflammation and lipid accumulation” signature (Dib et al., 2023) (Fig. 2). PLIN2^high^/TREM1^high^ LAMs also highly express of apoptosis and anti-proliferation genes (*SPINK1*, *G0S2*, and *BTG1*), suggesting a terminal inflammatory state (Mazitova et al., 2023; Zhu et al., 2022). Clinical studies have demonstrated a strong positive correlation between TREM1 and PLIN2 expression in atherosclerosis plaques, with both closely linked to CD68^+^ macrophages. Previous studies suggest PLIN2 as a core structural protein of LDs, which is mainly responsible for stabilizing LDs, inhibiting lipolysis, and maintaining cellular lipid storage and metabolic homeostasis (Wu et al., 2022). PLIN2^+^ and TREM1^+^ positive staining area overlaps substantially, and are significantly larger in symptomatic patients compared to asymptomatic patients (Dib et al., 2023). Patients with high TREM1/PLIN2 expression show elevated expression of inflammatory genes such as *TLR2*, *CCL2*, and *CXCL8,* have a significantly increased risk of cerebrovascular events, such as stroke and transient ischemic attack. This suggests that targeting PLIN2^high^/TREM1^high^ LAMs may reduce the risk of plaque complications (Dib et al., 2023).

Upon induction by soluble cholesterol or lipids *ex vivo*, foam cells downregulate cholesterol synthesis genes (*Cyp51* and *Hmgcr*) and upregulate cholesterol efflux genes (*Abca1* and *Abcg1*) and LXR genes (*Nr1h2* and *Nr1h3*) (Huang et al., 2022; Méndez-Barbero et al., 2013). In contrast, *Trem2*^−/−^ foam cells exhibit the opposite expression pattern, leading to intracellular cholesterol accumulation. In addition, TREM2 regulates CD36 to promote oxLDL uptake and suppresses ER stress to sustain foam cell survival (Kotla et al., 2014; Patterson et al., 2023; Shi et al., 2016). In HFD-treated myeloid-specific *Trem2* knock-out mice, plaque areas in the aortic arch and aortic sinus are significantly reduced, and this is associated with the role of TREM2 in promoting proliferation but inhibit apoptosis of CD68^+^ macrophages, thereby maintaining foam cell numbers (Chen et al., 2012; Parolini et al., 2014; Patterson et al., 2023). After an HFD, plasma levels of soluble TREM2 (sTREM2) are elevated (Örd et al., 2025). In humans, TREM2 protein expression is higher in carotid artery plaques than in normal arteries, and plasma sTREM2 levels are significantly higher in symptomatic patients than in asymptomatic individuals (Örd et al., 2025). This suggests that sTREM2 could serve as a biomarker for clinical diagnosis of plaque instability and disease severity.

Additionally, *SPP1*^high^ LAM subtype is identified in atherosclerosis carotid plaques, which is enriched in pathways related to extracellular matrix disassembly and lipid-mediated inflammatory response (Mocci et al., 2024). *SPP1*^high^ LAM subtype is more prevalent in symptomatic plaques and marks the transition from asymptomatic to symptomatic disease, whereas *TREM2*^high^ LAMs are in the progression from early to advanced lesions (Dib et al., 2023; Mocci et al., 2024).

Both TREM2^+^ macrophages and SPP1^+^ macrophages are also involved in other cardiovascular disease processes. In the late phase following myocardial infarction (MI), a dominant population of macrophages highly expressing *Trem2* emerges in the infarct zone (Jung et al., 2022). These *Trem2*^high^ macrophages highly express anti-inflammatory genes (*Il10*, *Tgfb1*, and *Alox15*), repair-related genes (*Spp1* and *Timp2*), and lipid metabolism-related genes (*Fabp5* and *Gpnmb*), possessing anti-inflammatory and pro-repair functions (Jung et al., 2022). sTREM2 improves post-MI cardiac systolic function, reduces fibrosis, and increases survival rates. In comparison, SPP1^+^ macrophages in cardiac tissue are enriched in LDs during the repair process of myocardial ischemia-reperfusion injury (MIRI) (Jiang et al., 2024; Kuppe et al., 2022; Palma, 2025). SPP1^+^ macrophages express a similar gene set to LAMs in atherosclerosis. Functionally, SPP1^+^ macrophages activate pathways involved in lipid phagocytosis and degradation, such as cholesterol metabolism and PPAR signaling, while downregulating MAPK pathway activity (Zhong et al., 2015).

In summary, lipid metabolic disorders and immune cell dysfunction are major causes of atherosclerosis, and LAMs in atherosclerotic plaques exhibit heterogeneity. The *TREM2*^high^ subtype is predominantly distributed in stable plaque regions and exhibits a homeostatic lipid-handling phenotype, whereas other subtypes such as PLIN2^high^/TREM1^high^ and *SPP1*^high^ are located in plaque rupture-prone areas, displaying dual characteristics of inflammation and lipid accumulation. These subsets of LAMs are involved in the progression of atherosclerotic plaques from early to advanced stages through multiple pathways, including mediating inflammatory responses, regulating lipid transport, and impairing plaque microenvironment stability. Targeting key LAMs, such as *TREM2*^high^ subtype, can reduce foam cell numbers and inhibit plaque progression. Furthermore, the expression levels of soluble TREM2 in plasma might be helpful for diagnosing disease severity and plaque stability. Therefore, understanding the functions of different LAM subsets in atherosclerosis might provide targets for the diagnosis or treatment of atherosclerosis and other related cardiovascular diseases.

## 7 Lipid droplet-accumulating microglias (LDAMs) in the aging brain

Growing interests also focus on the association between lipid metabolism disorders in the brain and neurodegenerative diseases. The brain contains abundant lipid components, which not only form the important structural basis of nerve cell membranes and myelin sheaths, but also regulate various physiological processes such as neural signal transmission and synaptic plasticity regulation for maintaining normal brain function. After entering old age, the overall metabolic function of the body including the brain changes, which is manifested as abnormal lipid synthesis, blocked degradation pathways, or decreased lipid transport efficiency to subsequently causes abnormal lipid accumulation in the brain (Chausse et al., 2021; Yin, 2023). Under aging, Alzheimer's disease (AD), and demyelinating diseases, microglia frequently adopt a foam-cell-like phenotype characterized by abundant LDs and cholesterol crystals, and such lipid-laden cells are collectively defined as LDAMs (Cantuti-Castelvetri et al., 2018; Marschallinger et al., 2020).

In the hippocampus of aged mice, LDAMs constitute more than 50% of the total microglial population, which display hallmark features of age-associated dysfunction and a pro-inflammatory phenotype, suggesting that LDAMs may represent a key detrimental microglial subtype in the aging brain (Linnartz-Gerlach et al., 2019; Streit et al., 2009). Accumulating evidence indicates that inflammation is a major driver of LD formation in microglia, and age-associated neuroinflammation triggers LDAM development (Marschallinger et al., 2020). LDs in hippocampal microglia of aged mice consists predominantly of triglycerides with negligible cholesterol esters (CEs) content, and distinct from hepatic LDs that are typically cholesterol-esters rich (Ioannou et al., 2017; Matsuzawa et al., 2007). When comparing with LD-low microglias, LDAMs from the hippocampus of aged mice secrete markedly higher levels of pro-inflammatory cytokines, including *CXCL10, CCL3, TNF-α*, and *IL-6 (*Marschallinger et al., 2020*)*. This finding indicates that LDAMs exist in a “primed” activation state, rendering them prone to hyperactivation upon external stimulation. Additionally, LDAMs display enhanced fatty acid β-oxidation activity, lysosome accumulation, and enrichment of signaling pathways associated with phagosome maturation and nitric oxide/reactive oxygen species. Stereotactic injection of myelin debris into the hippocampus of aged mice further demonstrated that LDAMs phagocytosed significantly fewer myelin particles than LD-deficient microglia.

## 8 LDAMs in the pathological brain

Dysregulated lipid metabolism under pathological conditions is a major driver of LDAM enrichment in brain tissues. In AD, the abnormal deposition of amyloid-β (Aβ) not only activates microglia but also induces lipid degradation in neuronal membranes, leading to the release of excess free lipids in the brain parenchyma. Short-term Aβ exposure can promote LD accumulation in wild-type microglia, whereas prolonged exposure saturates the microglial response to LDs and causes irreversible impairment of phagocytic function (Marschallinger et al., 2020). In 5 × FAD mice, microglia exhibit more LDs than those in wild-type mice, particularly in Aβ plaque-rich regions such as the hippocampal subiculum and cerebral cortex (Marschallinger et al., 2020; Prakash et al., 2025). These lipid-laden microglia display profound phagocytic defects (Muñoz Herrera and Zivkovic, 2022), and their abundance decreases with distance from Aβ plaques with the gradually normalized morphology and function (Prakash et al., 2025). A similar phenomenon has been observed in xenografted human iPSC-derived microglia in the brains of 7-month-old chimeric mice (Claes et al., 2021). Also, amyloid precursor protein (APP) knock-in mice show an increased proportion of LD-containing microglia, which is further exacerbated by HFD (Cho et al., 2022; O'Brien and Wong, 2011; Wu et al., 2025). Conditioned media derived from iPSC-based tauopathy neurons can similarly induce LD accumulation in primary mouse microglia, human iPSC-derived microglia as well as in BV2 cells (Li et al., 2024). LD accumulation impairs microglial phagocytosis of tau protein during AD progression and exacerbates neuroinflammation, as evidenced by increased transcription of *Il1b* and *Il6* in the cerebral cortex of male mice (Lee et al., 2023; Nie et al., 2025). Notably, unsaturated lipids released by neurons can be directly transferred to microglia, which then exhibit a pro-inflammatory phenotype characterized elevated TNF-α and IL-1α and impaired phagocytosis, representing the hallmarks of LDAMs (Li et al., 2024). Blocking LD formation using inhibitors of diacylglycerol acyltransferase 1 (DGAT1), which is a key enzyme catalyzing triglyceride synthesis, can partially restore the microglial phagocytic capacity for tau protein (Lee et al., 2023). Importantly, a higher proportion of LDAMs is detected in the brains of AD patients carrying the *APOE4/4* genotype (Amontree et al., 2023; Haney et al., 2024), and microglia from the hippocampus of human AD patients exhibit higher LD density and volume. Consistently, APOE4 knock-in into the J20 AD mouse model results in marked LD accumulation in hippocampal microglia.

Single-cell RNA sequencing analysis reveal that the transcriptome of disease-associated microglia (DAMs) surrounding Aβ plaques of the hippocampal subiculum exhibit similar genes as foam cells in human atherosclerosis, which significantly enrich genes such as *CD9* and *APOC1* (Claes et al., 2021; Fernandez et al., 2019; Mosher and Wyss-Coray, 2014; Wang et al., 2015). Notably, DAMs possess robust phagocytic activity, but LDAMs impaire phagocytic dysfunction (Marschallinger et al., 2020). The transcriptional signature of these LDAMs is different from that of DAM subsets in healthy, aged, and degenerating brains, suggesting their distinct functional phenotype.

In the cuprizone-fed mouse model of multiple sclerosis (MS), robust brain demyelination induces strong microglial activation followed by intracellular LD accumalations (Jewells et al., 2021; Plant et al., 2006). LDAM formation is accompanied by marked upregulation of genes associated with lipid transport and metabolism, including *Apoe*, *Apoc1, Lpl*, and *Ch25h* (Gouna et al., 2021; Poliani et al., 2015). Microglia co-expressing IBA1 and PLIN2 are readily detectable at the lesion site, and approximately 70% of these cells also express TREM2. In contrast, TREM2-deficient mice show near-complete absence of microglial foam-cell formation, and the number of IBA1^+^PLIN2^+^ cells is substantially lower than that in WT mice. These deficits are accompanied by massive accumulation of myelin debris at the lesion site, axonal dystrophy, and a marked reduction in oligodendrocyte numbers (Gouna et al., 2021; Poliani et al., 2015). These findings indicate that TREM2 promotes LD formation in microglia, and this process mounts a protective response of microglia to counteract cholesterol overload. Collectively, TREM2 is an essential mediator of LDAM formation and a key regulator of microglial activation of lipid metabolic programs in response to myelin injury. TREM2 deficiency abrogates the efficient phagocytosis and metabolism of myelin debris by microglia, thereby impeding remyelination (Gouna et al., 2021; Poliani et al., 2015). Notably, CE accumulation in TREM2-deficient cells is fully reversed by treatment with ACAT1 inhibitors or LXR agonists. In cholesterol acyltransferase (ACAT1) knockout mice, microglia at the lesion site fail to form LDs following demyelinating injury (Nugent et al., 2020; Shibuya et al., 2015), which instead increase IBA1^+^ cell density with the sustained innate immune inflammation and impaired remyelination post-injury (Nugent et al., 2020).

In summary, the brain is a lipid-rich organ, and relies on lipid metabolic homeostasis to maintain normal function. During the pathological processes of neurodegenerative diseases such as AD and MS, Aβ deposition, degradation of neuronal membrane lipids, and demyelination could induce LD accumulation in microglia, which are thus transformed into LDAMs characterized by abnormal LD accumulation. LDAMs accumulate in Aβ plaque-enriched regions in AD and in demyelinated lesions in MS, which trigger excessive neuroinflammation, impair the phagocytic function of microglia, and exacerbate the pathological deposition of Aβ and tau proteins as well as demyelinating damage. Studies have suggested that targeting DGAT1 in LDAMs reduces LD accumulation within microglia to restore their phagocytic function; targeting cholesterol metabolism-related enzymes reverses abnormal CE accumulation, while activating TREM2 signaling can promote microglial phagocytosis and metabolism of myelin debris and remyelination, inhibiting the pro-inflammatory activation features of LDAMs can alleviate excessive neuroinflammation. These strategies suggest that targeting LDAMs might be able to delay the progression of neurodegenerative diseases.

## 9 Similarities and differences of LAMs in different microenvironments

The formation of LAMs (referred to as LDAMs in the brain) in the four major organs including adipose tissue, liver, arteries, and brain, is primarily driven by lipid metabolic disorders. When systemic or local organ lipid uptake, synthesis, metabolism, or transport becomes imbalanced, leading to abnormal lipid accumulation, macrophages or their precursor cells are induced to enrich LDs, subsequently transforming into LAMs/LDAMs. Both LAMs and LDAMs are morphologically characterized by massive cytoplasmic LD enrichment and originate from cells of the monocyte-macrophage lineage (Cantuti-Castelvetri et al., 2018; Jaitin et al., 2019; Zernecke et al., 2023). Their differentiation processes are regulated by the local lipid microenvironment and cytokine signaling. LAMs/LDAMs in all organs exhibit an imbalance between dual functions: lipid metabolism regulation and inflammatory function (Cottam et al., 2022; Dib et al., 2023; Ganguly et al., 2024; Hildreth et al., 2021; Zhou et al., 2025a). Under physiological conditions or in the early stages of disease, LAMs/LDAMs maintain organ microenvironmental homeostasis by phagocytosing and clearing locally accumulated abnormal lipids and dead cell debris. Upon disease progression, their lipid-handling capacity becomes impaired; inflammation further accelerates LD accumulation within LAMs/LDAMs, and secrete large amounts of pro-inflammatory factors such as TNF-α, IL-1β, and IL-6, forming a positive feedback loop (Hildreth et al., 2021). This exacerbates lipid accumulation and inflammatory responses in LAMs/LDAMs, making them key drivers of disease progression. TREM2 is a core regulator shared by LAMs/LDAMs across all four organs (Ganguly et al., 2024; Jaitin et al., 2019; Örd et al., 2025; Zernecke et al., 2023; Zhou et al., 2025a), which regulates lipid phagocytosis, lipid metabolism, and inflammatory responses. Loss or knockout of TREM2 impairs the lipid-handling capacity and disrupts inflammatory regulation in LAMs/LDAMs, thereby exacerbating pathological damage in the respective organs.

LAMs/LDAMs in different organs exhibit differences in origin, distribution, molecular characteristics, and functional features. Regarding origin, in the adipose tissue of obese individuals, in the liver in metabolic disorder-related diseases, and in atherosclerotic lesions, LAMs primarily originate from monocytes (Daemen et al., 2021; Jaitin et al., 2019). In contrast, LDAMs in the brain of individuals with neurodegenerative diseases are transformed from resident microglia in response to lipid and inflammatory stimuli, rather than from the differentiation of infiltrating monocytes (Cantuti-Castelvetri et al., 2018; Marschallinger et al., 2020). In terms of distribution, LAMs in the adipose tissue of obese individuals are mainly localized to CLS surrounding dead adipocytes, with molecular expression differences between subcutaneous and visceral fat LAMs (Jaitin et al., 2019). In the liver in metabolic disorder-related diseases, LAMs are concentrated in fibrotic areas such as periportal regions and hCLS adjacent to steatotic hepatocytes (Xu et al., 2025a). In atherosclerotic lesions, the *TREM2*^high^ subtype is found in the stable fibrous cap of plaques, while the PLIN2^high^/TREM1^high^ subtype is localized to the lipid-rich core prone to rupture (Dib et al., 2023). LDAMs in the brains of individuals with neurodegenerative diseases specifically aggregate in pathological regions such as Aβ plaque-enriched areas and demyelinated lesions (Marschallinger et al., 2020). Regarding their molecular markers, CD9 expression in adipose tissue LAMs of obese individuals positively correlates with BMI and serves as a key marker for LAMs in visceral fat (Hill et al., 2018). Subcutaneous fat LAMs highly express TREM2 and CD248, while CD11b^+^ cells in visceral fat specifically express *LYVE1*, *TIMD4*, and *MRC1* (Reyes-Farias et al., 2025). Liver LAMs in metabolic disorder-related diseases are enriched for *Gpnmb*, *Ms4a7*, and *Cd63*, while showing low expression of KC-specific markers *Timd4*, *Clec4f*, *Marco*, and *Cd163*, enabling the distinction of liver LAMs from other macrophage populations (Daemen et al., 2021; Zhou et al., 2024). In atherosclerotic lesions, *Trem2*^high^ subtype can be further distinguished from *Gpnmb* ^high^ subtype versus *Slamf9* ^high^ subtype (Zernecke et al., 2023), while sTREM2 acts as a core marker of plaque instability and disease severity (Örd et al., 2025). In the brains of individuals with neurodegenerative diseases, LDAMs co-express IBA1, PLIN2 as well as CD9 and APOE, and their lipid composition differs significantly from that in other organs, with LDs primarily composed of triglycerides, in sharp contrast to the CE-rich characteristic of liver LAMs (Marschallinger et al., 2020).

Functionally, LAMs in different organs exhibit organ-specific biases, and this aligns with the physiological roles and disease pathological features of their resident organs. The core functions of adipose tissue LAMs are to regulate adipose tissue lipid homeostasis and adipose tissue inflammation and fibrosis in obese individuals (Hildreth et al., 2021). LAMs exacerbate obesity-related inflammation through monocyte recruitment, and LAMs also cause T-cell dysfunction via antigen presentation or cytokine secretion, resulting in obesity-related immune dysregulation (Hildreth et al., 2021). Liver LAMs in metabolic disorder-related diseases affect liver repair and hepatic inflammation and fibrosis. They can mitigate liver inflammation and fibrosis by clearing necrotic steatotic hepatocytes and phagocytosing toxic lipids, and also secrete HGF to promote liver repair. However, their functional imbalance exacerbates hepatic steatosis, inflammation, and fibrosis progression, representing a critical step in the transition from MASLD to MASH (Daemen et al., 2021; Li et al., 2025b). In atherosclerotic lesions, LAMs influence the formation and stability of atherosclerotic plaques. Lipid metabolism-dominant LAMs participate in forming the plaque lipid core, while inflammation-dominant LAMs secrete pro-inflammatory factors that disrupt vascular matrix integrity, promoting plaque progression, instability, and even rupture (Dib et al., 2023). Notably, *TREM2*^high^ macrophages in cardiovascular diseases such as myocardial infarction also exhibit anti-inflammatory and pro-repair functions, reducing fibrosis (Jung et al., 2022). In the brains of individuals with neurodegenerative diseases, LDAMs mediate neuroinflammation and impair phagocytic function. As a detrimental microglial subtype in aging and neurodegenerative diseases, LDAMs secrete large amounts of pro-inflammatory factors to exacerbate neuroinflammation and have an impaired phagocytic function, failing to effectively clear pathological substances, thereby aggravating neuronal damage and brain dysfunction (Prakash et al., 2025).

In conclusion, during systemic lipid metabolic disorders, different organs regulate the phenotypic transformation of monocyte-macrophage lineage cells to form LAM/LDAM subpopulations, and participate in the pathogenesis and progression of organ-specific diseases.

## 10 Therapeutic strategies targeting LAMs

Because LAMs are widely present in various tissues and disease microenvironments, and their expression of certain inflammatory factors and metabolic functional genes remains conserved, this endows LAMs as a therapeutic target (Xu et al., 2025b). Therapeutic strategies targeting LAMs or the reprogramming of their functions have been gradually developed for various diseases, such as cardiovascular disease, MASLD/MASH and AD. These immunotherapies target multiple biological processes, including lipid uptake, accumulation and efflux. Also, an increasing number of therapies are designed to target specific markers of LAMs and master genes involved in immune regulation. Novel agents that elevate the levels of anti-inflammatory cytokines have also been continuously developed. All these approaches aim to alter the functional state of macrophages and the immune microenvironment to alleviate diseases.

Canakinumab, a neutralizing antibody targeting IL-1β, which is mainly derived from LAM in atherosclerotic cardiovascular disease, has exhibited remarkable theraputic efficacy in atherosclerotic cardiovascular disease. It is the first drug to be clinically proven to reduce the recurrence of cardiovascular events in post-myocardial ischemia patients (Ridker et al., 2017). In LAMs, the NLRP3 inflammasome acts as a crucial upstream trigger for IL-1β production and has been well validated as a promising anti-inflammatory drug target. The NLRP3 inhibitor NT-0796 (ruvonoflast) blocks the activation of inflammatory signaling pathways in LAMs, exerts potential efficacy in reducing the production of CVD-associated inflammatory factors, and mitigates CVD risk in obese patients (Thornton et al., 2024). Anti-lipid therapy that suppresses LAMs' formation is also employed for the management of CVD, particularly atherosclerosis. Golocdacimab (MEDI6570), a monoclonal antibody antagonist targeting the oxLDL receptor OLR1/LOX-1, can inhibit the differentiation of inflammatory LAMs, ameliorate the function of endothelial and smooth muscle cells, reduce residual inflammatory risk, and lower IL-6 levels in post-myocardial ischemia patients (O'Donoghue et al., 2025; Vavere et al., 2023). Salvianolic acid B, a natural product targeting the alternative oxLDL receptor CD36, abrogates oxLDL uptake by LAMs, alleviates hypercholesterolemia and retards atherosclerotic progression and exerts a protective effect against MIRI (He et al., 2023). Engineered membrane-biomimetic liposomes loaded with retinoic acid can target lipid-enriched lesions, thereby inducing LAMs to engulf retinoic acid, upregulating ABCA1 and ABCG1 to promote cholesterol and lipid efflux, shifting LAMs toward an anti-inflammatory phenotype, and retarding atherosclerotic progression (Cai et al., 2026). ACAT1 is involved in cholesterol esterification and intracellular accumulation in macrophages, and the small-molecule ACAT1 inhibitor pactimibe has been shown to mitigate systemic inflammation and improve vascular endothelial function in patients with hypercholesterolemia. Furthermore, phase 2 clinical trials of pactimibe for the treatment of coronary artery disease (CAD) and familial hypercholesterolemia (FH) have been completed (Meuwese et al., 2009). Pharmacological inhibition of TREM1 by a peptide inhibitor named nangibotide attenuates the progression of experimental atherosclerosis (Joffre et al., 2016).

Thiazolidinedione agonists targeting PPAR-γ, a core transcription factor governing lipid metabolism in LAMs, can reprogram LAMs from a pro-inflammatory toward a protective phenotype (Kumar et al., 2018). In patients with MASLD, saroglitazar therapy improves lipid profiles independent of statin administration (Siddiqui et al., 2023). Saroglitazar not only exerts beneficial effects on MASLD-related liver pathology, but also mitigates cardiovascular risk in patients with MASLD (Moka et al., 2025). In addition to PPAR-γ, LXR-α/β are key transcription factors for lipid-associated macrophages. Because LXR not only influences the expression of metabolic genes but also possesses anti-inflammatory functions, drug development targeting LXR encompasses two directions: inhibitors or agonists. Inhibitors are suitable for metabolic diseases, while agonists are promising in onco-therapy. For example, TLC-2716 is a first-in-class oral LXR inhibitor currently being developed for the treatment of dyslipidemia, hypertriglyceridemia, and MASLD, which is now undergoing a phase 2a clinical study (Li et al., 2026); while the LXR agonist Abequolixron (RGX-104) has recently completed in phase 1 clinical trials against oncology (Tavazoie et al., 2018).

Beyond targeting these core transcription factors, activation of the macrophage-expressed TREM2 receptor can also reprogram LAM functions, reduce inflammatory factor expression, and enhance efferocytosis and cholesterol efflux (Deczkowska et al., 2020; Nicolás-Ávila and Hidalgo, 2023). TREM2-targeting antibody agonists, including iluzanebart (VGL-101) and VHB937, show promising therapeutic efficacy in phase 2 clinical trials for the treatment of diseases such as AD and ALS (Colonna and Holtzman, 2025; Long et al., 2024). In addition to these antibody agonists, small-molecule TREM2 agonists also exhibit promising translational potential. These small molecules can cross the blood-brain barrier, do not bind to sTREM2, and maximize TREM2 receptor activation and microglial function, thereby augmenting the therapeutic potential for neurodegenerative diseases (Yang et al., 2025). TREM1, a paralog of TREM2, acts as a surface marker for inflammatory LAMs, and its expression correlates with atherosclerotic complications including cerebral ischemic events (Lavine, 2023).

**Fig. 3:**
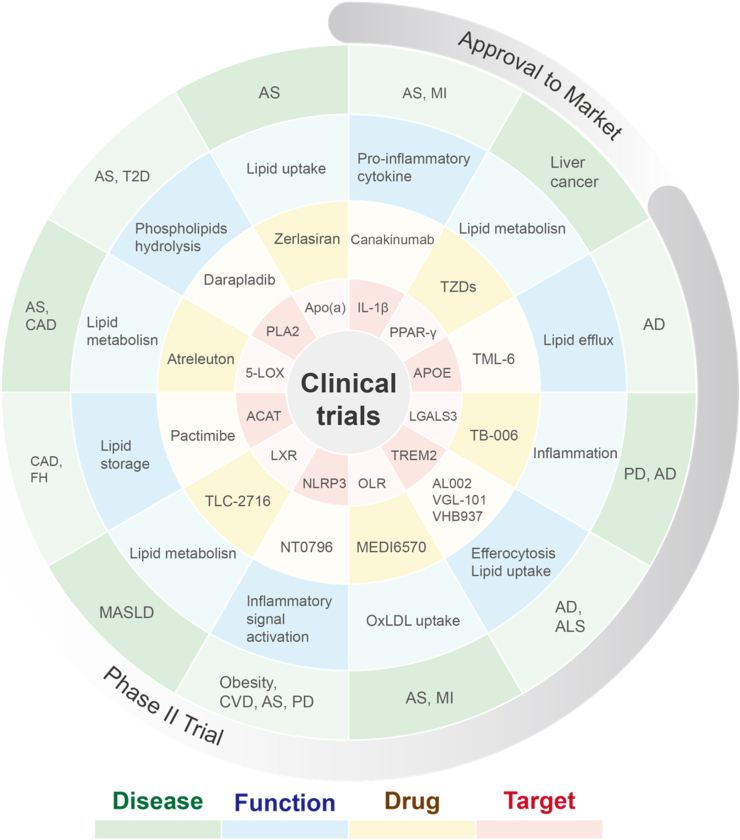
**Clinical translation of LAM-related targets** AD: Alzheimer's disease. ALS: amyotrophic lateral sclerosis. Apo(a): apolipoprotein(a). AS: atherosclerosis. CAD: coronary heart disease. CVD: cardiovascular disease. FH: familial hypercholesterolemia. MASLD: metabolic dysfunction-associated steatotic liver disease. MI: myocardial infarction. OxLDL: oxidized low-density lipoprotein. PD: Parkinson's disease. T2D: type 2 diabetes. TZDs: thiazolidinediones.

In summary, the current therapeutic strategies and research advances targeting LAMs are predominantly focused on three core aspects, including inflammatory suppression, lipid metabolic regulation, and functional polarization of macrophages (Fig. 3). Additionally, epigenetic regulation might also be potential treatment against macrophage inflammatory responses and lipid metabolism. For instance, small non-coding RNAs, represented by miR-21, miR-155, miR-33, and miR-144-3p, exhibit considerable translational potential for clinical application. In the advanced stages of atherosclerosis, therapeutic local delivery of miR-21 to carotid plaques or systemic inhibition of miR-155 can abrogate macrophage-mediated secretion of inflammatory mediators and upregulate IL-10 expression. Anti-miR-33 and anti-miR-144-3p therapies further promote ABCA1-and ABCG1-mediated cholesterol efflux from macrophages, thereby alleviating vascular lipid accumulation and associated inflammatory responses (Peng et al., 2022; Price et al., 2017). Furthermore, exosomes and nanovaccines are in continuous development (Li et al., 2025a). LAM-based therapeutic strategies are tailored to the specific disease context. For instance, LAMs drive malignant tumorigenesis and progression via their immunosuppressive phenotype, whereas they mediate protective effects in steatohepatitis and septic myocarditis (Ganguly et al., 2024; Nicolás-Ávila and Hidalgo, 2023). Therefore, therapeutic approach, whether to activate LAMs and augment their abundance, or to block and deplete them, depends on the diseases.

## 11 Conclusion and perspective

Recent studies to discover and study LAMs have expanded our understanding of macrophage heterogeneity and functional plasticity, highlighting the crucial role of LAMs at the interface of lipid metabolism, immune responses and inflammation-related diseases. With rapid advances in high-throughput and spatially resolved technologies, including single-cell and spatial transcriptomics, LAM-related research has evolved from initial subset identification and basic phenotypic characterization toward mechanistic dissection and translational exploration. Through regulating lipid clearance, inflammation, and tissue remodeling, LAMs actively shape disease initiation and progression, which offers new strategies for the treatment of metabolic–immune-related diseases. Future studies on LAMs may focus on three major directions. First, systematically dissect the heterogeneity of LAMs across tissues, metabolic disease contexts, and disease stages to clarify their subtype-specific molecular, metabolic, and functional features. Current research is largely confined to single diseases or tissues, leaving the cross-disease and cross-tissue comparisons and the dynamic evolution of LAMs subtypes incompletely understood. Integrative single-cell, spatial, and multi-omics approaches are critical for mapping LAM subtypes, identifying subtype-specific markers, and enabling improved disease diagnosis and patient stratification. Second, further elucidation of the regulatory crosstalk among LAMs, lipid metabolism, inflammatory responses, and neighboring cellular populations is needed. LAMs interact with other immune cells and parenchymal cells, and the underlying mechanism especially those involving epigenetic modifications and non-coding RNAs, remain largely unresolved. Identifying specific lipid metabolites that act as signaling molecules within LAMs and determining how these metabolites modulate immune function, represent important priorities. Combining gene editing approaches and systemic animal models will be crucial to overcoming these bottlenecks and clarifying the molecular pathogenic mechanisms by which LAMs drive metabolic diseases. Third, accelerating the development of precise and safe LAM-targeted therapeutic strategies is critical for clinical translation. Nevertheless, although targeting LAM core molecules such as TREM2 and CD36 has shown potential in preclinical models, current LAM-related diagnostic markers lack sufficient sensitivity and specificity for clinical use. Future efforts should focus on developing multi-marker diagnostic platforms, subtype-specific therapeutic strategies, and approaches to reprogram or reeducate pathogenic LAMs toward protective phenotypes in advanced disease stages. Integration of nanotechnology, gene-editing tools, and advanced delivery systems may further enhance targeting efficiency and translational feasibility.

To conclude, LAMs act as a central cellular hub connecting metabolic dysregulation and immune inflammation, and represent a promising frontier in metabolic disease research. Continued investigation into LAM heterogeneity, regulatory networks, and intercellular crosstalk will help overcome current therapeutic bottlenecks. Such efforts, combined with ongoing technological innovation, hold the potential to identify precise intervention targets and diagnostic strategies for the clinical management of obesity, MASH, atherosclerosis, and age-related neurodegenerative diseases.

## CRediT authorship contribution statement

**Yuxiao Zheng:** Writing – review & editing, Writing – original draft, Visualization, Conceptualization. **Dezhen Tu:** Writing – review & editing, Funding acquisition. **Chen Dong:** Writing – original draft, Conceptualization. **Haiqing Zhao:** Writing – review & editing. **Hongyan Wang:** Writing – review & editing, Supervision, Funding acquisition, Conceptualization.

## Declaration of competing interest

H. Wang holds the position of Associate Editor for *Cell Insight* and is blinded from peer review and decision making for the manuscript. The rest of the authors declare that they have no known competing financial interests or personal relationships that could have appeared to influence the work reported in this paper.
